# Leaderless Medicine

**Published:** 1894-03-17

**Authors:** 


### The Hospital

An Institutional Journal of

The Medical Sciences and Hospital Administration, with

AN EXTRA NURSING SUPPLEMENT.

Vol. XV.—No. 390.] [March 17, 1894.

### Leaderless Medicine.

Medicine is an instrument for promoting human energy and happiness, and for curing or minimising the physical ills which affect the human species. Those who recognise in medicine an instrument of this nature cannot but desire to see it so employed that it shall promote human energy and happiness, and cure or minimise the physical ills of mankind in a maximum degree. A critical observation of the experimental, literary, and practical fields of modern medicine leads to the conclusion that a great deal of the knowledge, and even of the clinical skill of the period, is running to absolute waste. The few have knowledge, the many have not knowledge; the few are keen and skilful experts, the many are not keen, are not skilful, are not experts, the few are definite in their knowledge and methods—almost as definite as the mathematician or the engineer, the many are vague, uncertain, feeble, and untrustworthy.

There are many circumstances at the present time which call attention to and emphasise this invertebrate condition of things. One such circumstance is the effort now being strenuously made to create a teaching university for the metropolis of Great Britain; another is the increasingly obvious fact that in the late Sir Andrew Clark the medical profession lost a man who, if he had been placed in the position of a teacher and a leader instead of in that of a mere practising physician, would in all probability have pushed forward the whole profession to a position at least twenty years in advance of its present and actual standpoint. Dr. Sheridan Delepine, who, as is well known, was for many years scientific assistant and colleague of the late President of the College of Physicians, has brought to a focus in the *Journal of Pathology* some of the scientific attainments, originalities of view, wide outlooks, great educational purposes, and splendid enthusiasms of his illustrious colleague. Dr. Delepine's paper will come as a revelation to many, especially to those young men of limited reading and narrow laboratory outlook who have so confidently assured the world that Sir Andrew Clark was no doubt a successful fashionable physician, but that "he did nothing of any permanent value to advance the science and clinical competency of medicine."

Sir Andrew Clark had the endowments of an investigator, a teacher, and a leader of men. The interests of medicine as a science and an art demand above all things men who combine in their own persons the characters of scientific investigators, able expositors, and great leaders. Such men are as rare as Shakespeares or Miltons. Not once in a century does the medicine of the whole world produce one such man. And yet when he is produced the only use we can make of him is to put him into a consulting-room in a fashionable West-end square to earn fifteen thousand a year by daily drudgery. That may have been a fit and proper use to put a man of Sir Andrew Clark's powers from the point of view of mere money-making; but from the standpoint of medical and scientific progress and of the culture and efficiency of the whole medical profession it was a sheer waste of the finest gold of medical and scientific capacity.

Dr. Delepine proves conclusively that as a scientific investigator the late President of the College of Physicians was possessed of endowments of the highest order. "It is not generally known," he says, "that as early as 1854 Andrew Clark gave a description of the minute anatomy of the lungs much in advance of that given not long before by no less an authority than Kölliker, The point which chiefly attracted his attention was the relation of the alveoli to the air passages. . . . On this point his views have been gradually more and more confirmed . . . they were more correct than those of the illustrious German histologist." A single passage from one of Clark's published papers well illustrates the breadth, strength, and clearness of his intellect, and incidentally also the fact that twenty years ago he was greatly in advance, so far as philosophical insight was concerned, of many men of no mean standing who belong to the present day. "Men," he says, "at the outset of their inquiries, adopting the provisional formula of Laennec, get infatuated by its simplicity, and persist in declaring it to be absolute. Every cheesy lump is a tubercle. Tubercle is a new growth, resulting always from a special constitutional vice, and never from a neglected cold, an unabsorbed pneumonic deposit, or a fibroid invasion of the lung. Pulmonary phthisis is simply the suppurative disintegration of the tubercular deposit. However different may seem the anatomical changes affecting and accompanying the destruction of the lung in this disease—grey lumps or yellow, circumscribed or diffused, acute or chronic, fibrous or tubercular, pneumonic or hæmorrhagic—they are all but varieties the one of the other . . . For other organs there are, it is admitted, various agents of destruction; for the lung, however, there is practically none but tubercle. Much that is plausible can be urged in support of these views; and it requires rare freedom from traditionary influences, much careful observation, and the closest thought, to become completely convinced of their fallacy." Nevertheless those views in their essence are now known to be fallacious. The merit of Clark was that he knew them to be fallacious when practically all other physicians of weight and name were declaring them to be the truth and the whole truth; and whosoever differed from them did so at his peril. In like manner Clark showed a mixed faith and scepticism in his attitude towards bacteriology worthy of the most judicial intellect and the broadest philosophical temper. "He entertained towards modern bacteriology," says Dr. Delepine, "at times warm feelings of admiration, though at others he felt that bacteriologists were too often wanting in clinical or even pathological knowledge, and for that reason he often distrusted their results." Surely herein every competent physician will entirely agree with him. The mention of some of the various subjects on which Clark wrote may complete the barest outline of the life-work of a physician whose mind was interested in every question of medicine, and, indeed, in every question of man. He contributed papers on pathology, on the lungs, the alimentary canal, the genito-urinary organs, the circulatory organs, on nervous diseases, on alcohol, and on general subjects. Whenever he wrote or spoke, he wrote and spoke from adequate knowledge, with philosophical breadth and clearness, and with all the enthusiasm of personal conviction.

Now such a man as this, we maintain, was designed by nature, not to be the drudge and slave of one single consulting-room, but the inspiration and the illumination of many consulting-rooms. His natural destination was that of the teacher's platform. Medicine ought to have been able to offer him a place where generation after generation of young men might have seen with his eyes, studied with his zeal, worked with his methods and been animated, not for a year, but for a lifetime, by his spirit. Medicine had no such place to offer him. He was driven by force of uncontrollable circumstances to the consulting-room, and so, like another man of equal or even greater genius, he gave up to a handful of non-professional clients what was meant for the inspiration of the whole medical world. The causes which operated to take Andrew Clark away from teaching and to make him the drudge of a fashionable consulting-room are still in full operation. To-day medicine offers nothing to her teachers, nothing that is but starvation and insignificance. Because medicine can offer nothing to her teachers, she can secure for herself no leaders; and because she has no leaders of recognised authority her painfully-acquired knowledge lies, so to speak, by the wayside in wasting heaps; and the rank and file of the profession are backward in knowledge and deficient in skill. There is now an opportunity of creating positions of authority and leadership in the proposed new University for London. If the profession, as a body, has any collective intellect at all it will leave no stone unturned until positions which the ablest men in medicine may be proud to fill for a lifetime shall be ready for their ambition and choice.

